# Dr. Samuel Staniland’s New Mode of Accelerating Labour

**Published:** 1842-06-18

**Authors:** Samuel Staniland


*New Mode of Accelerating Labour.* By SAMUEL STANILAND.—Being a midwifery pupil of Dr. Blundell, I of course carried his valuable instructions with me into practice, and one (not the least—viz., that " meddlesome midwifery is bad") had much influence on my conduct when called to attend the parturient female. At first the unnecessary calls which were made for my attendance, and the frequently slow progress of labour in others, where the true expulsatory efforts had commenced, together with the circumstance of being urged, on various occasions, to assist labour if possible, made me reflect upon the nature of the physiological action of the uterus, and the expulsatory action of the abdominal muscles. Much thought on the subject, connected with the latter action on the rectum, bladder, stomach, and lungs, when irritating substances need expulsion, led me to the consideration of the process of labour, which appeared to me to be an analogous action, and I at length found the principle of imitation of the natural stimulant power, which I have now for ten years carried into very successful operation, not only with a saving of much suffering to the patient, but an immense saving of time to myself.

When I first practised it, I was not so conversant with the physiology of the animal economy (except by book and school knowledge) as at present; yet, having studied Nature's designs in her various sympathies, the principle of excitation and reflex influences came to my assistance, and taught me that as the bladder, when distended with urine, and the rectum distended with fæces, call upon the abdominal muscles to expel their contents, so would an excitement, similar to that of the head of the child, or the unbroken membranes on the vagina in their passage, promote, under proper circumstances, the natural efforts, and lead to a more speedy and equally safe labour.

The principle being given, what is the practice, and under what circumstances should the operation be performed?—for I desire never to forget Dr. Blundell's valuable maxim.

The practice consists simply in imitating the influence of the child's head or membranes on the natural passages, and thus producing a reflex and wholesome contraction of the abdominal muscles (I say nothing of the independent action of the uterus) by introducing the forefinger, or fore and second fingers, as far as the point of the os coccygis, and passing them along the whole surface downwards of the vagina, so as to give the sensation of distension, not pain, just enough to excite the required action, and give new and more vigorous impulse, even after hot fluids, stimulants, ergot, &c., have been tried in vain.

What are the circumstances, then, which warrant its application?

1. In cases of first labour, where the os uteri is fairly dilated, the head of the child has been in the passage from eighteen to twenty-four hours, the patient much fatigued, and the pains, without assistance, comparatively ineffectual, the womb acting.

2. In cases after the first child, where the passages are rather confined, or the head of the child unyielding, although in the passage, and the natural efforts unavailing, especially when warm fluids and stimulants have been tried without benefit.

3. Where there is much rigidity of the perineum, on which the head rests, but the natural efforts either greatly exhausted or, the perineum unyielding; the practice here serves two purposes,—viz., dilatation and stimulation.

Lastly. Where the womb and abdominal muscles, after a severe labour, sink into a collapsed state, and are indisposed to do more work, and the placenta, though in the upper part of the passage, does not excite the abdominal muscles to action. With this principle at my command, I have never seen such a thing as the old retained placenta in any form, and much doubt whether, with it, I ever shall.

There are other minor circumstances which it will be necessary to apply, but the observant practitioner will readily find them out when conversant with the application of the principle, and the above will be sufficient to guide him in most cases. If, however, with these directions, there should be any doubt on his mind, I would say, rather wait the result of the old practice than urge assistance when unnecessary or "meddlesome."

I shall feel much pleasure in hearing from any gentleman, either personally or through your Journal, the results of their practice, and the more so if they will favour us with new aphorisms for its general application.

Dr. Marshall Hall will at once perceive in this principle a practical application or illustration of the excito-motory nervous sensation; but it was not in consequence of his suggestion that the principle was applied by me, but after a study of the great sympathetic nerve, for I had practised it nearly five years before I read his excellent work.—*Provincial Med. and Surg. Journ.* March 26th, 1842.

